# Morphological and microsatellite DNA diversity of Djallonké sheep in Guinea-Bissau

**DOI:** 10.1186/s12863-021-01009-7

**Published:** 2022-01-07

**Authors:** Guiguigbaza-Kossigan Dayo, Isidore Houaga, Martin Bienvenu Somda, Awa Linguelegue, Mamadou Ira, Maurice Konkobo, Bacar Djassi, Joao Gomes, Mamadou Sangare, Bernardo Cassama, Chia Valentine Yapi-Gnaore

**Affiliations:** 1 Centre International de Recherche-Développement sur l’Elevage en zone Subhumide (CIRDES), Bobo-Dioulasso, 01 BP 454 Burkina Faso; 2 Institut du Sahel (INSAH/CILSS), BP 1530 Bamako, Mali; 3 Current address: Centre for Tropical Livestock Genetics and Health (CTLGH), Roslin Institute, University of Edinburgh, Edinburgh, UK; 4 Université Nazi BONI (UNB), Bobo-Dioulasso, 01 BP 1091 Burkina Faso; 5 Direction Générale de l’Elevage (DGE), BP 26 Bissau, Guinea-Bissau

**Keywords:** Sheep, Morphological diversity, Population structure, Microsatellite DNA, Guinea-Bissau

## Abstract

**Background:**

The present study aimed at characterizing the Djallonké Sheep (DS), the only local sheep breed raised in Guinea-Bissau. A total of 200 animals were sampled from four regions (Bafatá, Gabú, Oio and Cacheu) and described using 7 visual criteria and 8 measurements. These parameters have been studied by principal components analysis. The genetic diversity and population structure of 92 unrelated animals were studied using 12 microsatellite markers.

**Results:**

The values of quantitative characters in the Bafatá region were significantly higher than those obtained in the other three regions. A phenotypic diversity of the DS population was observed and three genetic types distinguished: animals with “large traits” in the region of Bafatá, animals with “intermediate traits” in the regions of Gabú and Oio and animals with “small traits” in the Cacheu region. The hair coat colors are dominated by the white color, the shape of the facial head profile is mainly convex and the ears “erected horizontally”. Most of the morphobiometric characteristics were significantly influenced by the “region” and “sex of animals”.

The average Polymorphism Information Content (PIC) of 0.65 ± 0.11 supports the use of markers in genetic characterization. Gabú subpopulation had the highest genetic diversity measures (*He* = 0.716 ± 0.089) while Cacheu DS subpopulation presented the smallest (*He* = 0.651 ± 0.157). Only Gabú and Bafatá subpopulations presented significant heterozygote deficiency across all loci indicating possible significant inbreeding. Mean values for *F*_*IT,*_ *F*_*ST*_*, F*_*IS*_ and *G*_*ST*_ statistics across all loci were 0.09, 0.029, 0.063 and 0.043 respectively. The overall genetic differentiation observed between the four DS subpopulations studied was low. Bafatá and Gabú are the most closely related subpopulations (D_S_ = 0.04, genetic identity = 0.96) while Bafatá and Cacheu were the most genetically distant subpopulations (D_S_ = 0.14, genetic identity = 0.87). Using Bayesian approach, the number of K groups that best fit the data is detected between 2 and 3, which is consistent with the morphological analysis and the factorial analysis of correspondence.

**Conclusions:**

The molecular results on DS population of Guinea-Bissau confirmed the ones obtained with morphological analysis. The three genetic types observed phenotypically might be due to a combination of the agro-ecological differences and the management of breeding rather than genetic factors.

**Supplementary Information:**

The online version contains supplementary material available at 10.1186/s12863-021-01009-7.

## Background

Livestock is an important source of income, livelihoods, nutrition and food security, as well as resilience in sub-Saharan Africa [1]. In the Republic of Guinea-Bissau, like other West African countries, the economy is dominated by the primary sector (agricultural production) with a contribution of approximately 62% to the Gross Domestic Product (GDP) and agriculture contributes to creating around 95% of jobs [2]. Livestock sector represents the second economic activity after agricultural crops and contributes to 17% of national GDP and 32% of agricultural GDP [3]. In its various forms, livestock occupies 72% of the rural population through multiple functions (economic, social, reserve and savings capital, labor power and improving soil fertility) [4].

The livestock population in Guinea-Bissau is relatively large, very diverse and includes cattle, goats, sheep, pigs, poultry and other animal species [5]. The farming system practiced is of extensive agro-pastoral type with certain specificities depending on the region.

Despite the socio-economic importance of livestock sector in Guinea-Bissau, the animal genetic resources are under-exploited and less valued. In recent years, the contribution of the livestock sub-sector to GDP decreased to 3.5% of national GDP and 7.8% of agricultural GDP [4]. The authors explain this decline by an absence of effective and sustainable strategies for the management of animal genetic resources despite the great potential and assets available to the country. The development of an efficient management strategy of domestic animal genetic resources in Guinea-Bissau requires the characterization and inventory of these genetic resources in order to guide decision-making [6, 7].

In Guinea-Bissau, small ruminants are important in animal husbandry and play a social and nutritional role. Indeed, they are commonly used as a source of protein during social and religious ceremonies (birthday celebrations, baptisms, funerals, weddings) and constitute a savings strategy [8]. They are among the most dominant domestic animal species in the east and north of the country. Djallonké sheep (DS) represents the main local sheep breed of Guinea-Bissau. Despite their appreciation (hardiness, resistance, trypanotolerance, prolificacy and sexual precocity), information on the phenotypic characteristics is very little documented while the molecular characterization has never been done. The goal of the present study was to improve the knowledge on the local sheep genetic resources of Guinea-Bissau in order to develop sustainable strategies for their development. The specific objectives of this study were to determine the morphobiometric characteristics and to evaluate the genetic diversity of the local DS population in four regions in Guinea-Bissau.

## Results

### Morphological characterization

#### Quantitative characters

Basic statistics of quantitative traits in DS subpopulations in the four regions are presented in Table 1.

**Table 1:** Descriptive statistics of the morphological traits of the four Djallonké Sheep subpopulations studied

Characters		Bafatá	Cacheu	Gabú	Oio	est (***P-***value)	All subpopulations
Chest Girth (cm)	min- max	60–82	56–89	56–93	59–80	KW S (*P <* 0.001)	*56–93*
	Means ± SD	72.80 ± 4.68a	67.40 ± 6.14b	67.22 ± 6.58b	69.35 ± 4.83b		*69.76 ± 6.02*
Chest Depth (cm)	min- max	32–43	29–39	27–45	23–51	KW S (*P <* 0.001)	*23–51*
	Means ± SD	38.05 ± 2.41a	33.84 ± 2.34b	33.97 ± 3.67b	35.15 ± 4.42b		*35.72 ± 3.76*
Height at withers (cm)	min- max	48.4–74.4	46.4–58.4	46.4–62.4	46.4–62.4	KW S (*P <* 0.01)	*46.4–74.4*
	Means ± SD	55.65 ± 4.16a	53.4 ±2.85b	53.23 ± 3.77b	54.67 ± 3.44ab		*54.46 ± 3.89*
Body Length (cm)	min- max	49–70	45–63	50–72	52–64	ANOVA S (*P <* 0.001)	*45–72*
	Means ± SD	60.75 ± 4.51a	53.16 ± 4.13b	57.50 ± 4.41c	57.80 ± 2.88c		*58.23 ± 4.78*
Ear Length (cm)	min- max	8–13	7–9	8–13	9–13	KW S (*p* < 0.001)	*7–13*
	Means ± SD	10.23 ± 0.95a	8.16 ± 0.62b	10.15 ± 1.02 ac	9.73 ± 0.78c		*9.85 ± 1.12*
Tail Length (cm)	min- max	25–45	19–29	19–33	20–31	KW (*P <* 0.001)	*19–45*
	Means ± SD	32.00 ± 3.29a	23.88 ± 2.89b	26.73 ± 2.79c	25.28 ± 2.77bc		*28.06 ± 4.36*
Horn Length (cm)	min -max	6–19	15–23	9–26	2–22	KW (NS)	*2–26*
	Means ± SD	13.81 ± 3.85	18.33 ± 4.16	14.07 ± 5.11	10.89 ± 6.9		*13.60 ± 5.21*
Interval Length between the roots of the two horns (cm)	min- max	7–13	4–6	5–10	4–8	KW (NS)	*4–13*
	Means ± SD	10.47 ± 3.56	4.67 ± 1.15	7.33 ± 1.45	6.22 ± 1.48		*8.24 ± 2.52*

*SD* Standard Deviation, *min* Minimum, *max* Maximum, *S* Significant, *NS* Non significant, *KW* Kruskal-Wallis test, *ANOVA* Analysis of variance

**Fig. 1:**
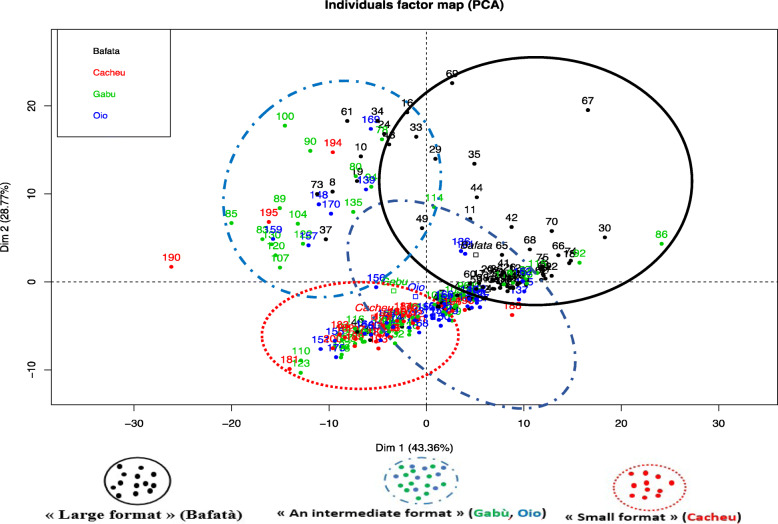
Principal components analysis to study the population structure

The values of the Chest Girth (CG), Chest Depth (CD), Height at withers (HW), Ear Length (EL) and Tail Length (TL) in Bafatá subpopulation were significantly higher (KW test, *P <* 0.001) than those of Cacheu, Gabú and Oio regions. In addition, the animals from the Bafatá region had significantly higher Body Length (BL) (ANOVA, *P <* 0.001) than those from other regions. The “region” or “location” had a significant effect on the most of the quantitative body characters of the DS in Guinea-Bissau as presented in Table 1, excepted the following traits: “Horn Length” and “ Interval Length between the roots of the two horns”. Three genetic types of DS were distinguished in the four regions: the type with “large traits” for animals in the Bafatá region, the type with “small traits” for animals in Cacheu region and the type with “intermediate traits” for animals in the Gabú and Oio regions. The three genetic types were revealed by the Principal Components Analysis (PCA). The Fig. 1 shows the individuals of Bafatá (black), the individuals of Cacheu (red) and a more heterogeneous population in Gabú (green) and Oio (blue).

In the studied population, 81.5% of animals sampled were females against 18.5% of males and all were 2 to 4 years. A sexual dimorphism was observed for some body parameters. Female animals had higher BL, CG and CD than their male counterparts (Table 1). Contrariwise, male animals had higher Horn Length and Interval Length between the roots of the two horns than the females.

### Qualitative characters by region

**Fig. 2:**
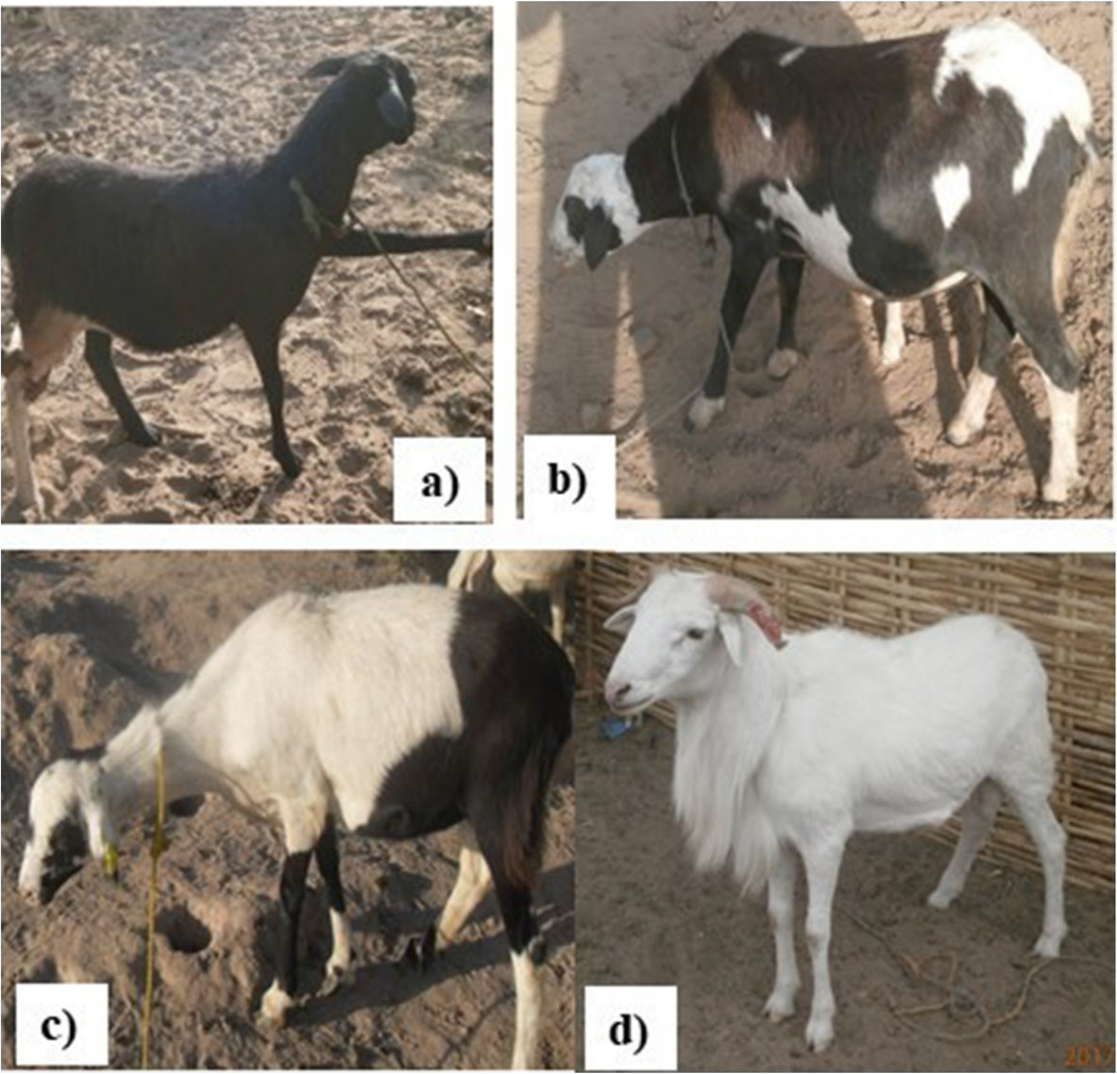
**a** Uniform black with tan belly; **b** Spotted/pied; **c** Patchy white-black with badger face; **d** Uniform white (PROGEVAL, 2017)

Values of the qualitative characters of the DS by region are presented in Table 2. In the Gabú, Cacheu and Oio regions, the uniform white body coat color was predominant with 81.67, 76.00 and 50.00% respectively. In Bafatá, the eumelanin-black color with tan belly (49.33%) and the uniform white (37.33%) and then the pheomelanin-brown and tan belly (13.33%) were mainly found. The uniform red/fawn was not observed in this study. The type of melanin observed had a significant link with the region (Chi^2^-test, *P <* 0.001). For the coat color patterns, the uniform white pattern characterized the DS in Gabú, Cacheu and Oio regions, while in Bafatá region the patchy (white-black or white-red/fawn) and the spotted (white color with some black or red/fawn spots without regular distribution) patterns were mostly observed in the proportions of 37.33 and 33.33%, respectively. The patchy pattern with badger face, plain black/brown, black/brown and tan white belly patterns were observed in the Bafatá and Oio regions. Figure 2 illustrates the coat color patterns of black/brown and tan, spotted pattern, patchy (white-black/white-fawn) and uniform white color.

**Table 2:** Distribution of the qualitative traits of Djallonké Sheep

Qualitative traits	Bafatá	Cacheu	Gabú	Oio	Chi^**2**^-test
**Coat color patterns (%)** Patchy (white-black/white-fawn)	13.33	4.00	3.33	20.00	
Patchy with badger face	9.33	4.00	1.67	0.00	
Uniform white	37.33	76.00	81.67	50.00	
Uniform black/brown	1.33	0.00	0.00	5.00	
Black/brown with tan belly	5.33	0.00	0.00	2.50	
Spotted of white and black / red/fawn	33.33	16.00	13.33	22.50	S (*P <* 0.001)
**Types of melanin pigments (%)**					
Absence of pigment (Uniform white)	37.33	76.00	81.67	50.00	
Pheomelanin	13.33	8.00	3.33	7.50	
Eumelanin	49.33	16.00	15.00	42.50	S (*P <* 0.001)
**Horn presence (%)**					
Presence	28	12	25	22.5	
Absence	72	88	75	77.5	NS (*P >* 0.05)
**Horn shape and orientation (%)**					
Lateral and straight horns	42.86	33.33	80.00	55.56	
Prismatic or corkscrew	0.00	0.00	0.00	11.11	
Backward spiral horns	23.81	0.00	6.67	0.00	
Spiral horns facing forward	9.52	66.67	13.33	33.33	
Stumps	23.81	0.00	0.00	0.00	*P =* 0.056
**Ear orientation (%)**					
Erect horizontally	97.33	100.00	100.00	100.00	
Semi-pendulous	2.67	0.00	0.00	0.00	NS (*P >* 0.05)
**Facial (chamfer) profile (%)**					
Convex	89.33	96	100	82.5	
Straight	10.67	4	0	17.5	S (*P <* 0.01)

*S* Significant, *NS* Non significant

In Cacheu, Gabú and Oio regions, all the animals carried horizontally erected ears, while 2.67% of the animals in Bafatá region had semi-pendulous ears. The facial (chamfer) profile of animals was predominantly convex. The straight shape was also observed in Bafatá (10.67%), Cacheu (4.00%) and Oio (17.50%).

The different horn shapes and orientations observed in the DS are presented in Table 3. No significant difference was observed between the regions (*P =* 0.056).

**Table 3:** Effects of sex on significant morphological characters

Characters	Attributes	Females	Males	Chi^**2**^-test
**Facial (chamfer) profile (%)**	Convex	90.18	100	
	Straight	9.82	0	S (*P <* 0.01)
**Horn presence (%)**	Presence	6.75	100	
	Absence	93.25	0	S (*P <* 0.001)
**Horn shape and orientation (%)**	Lateral and straight horns	45.45	59.46	NS
	Prismatic or corkscrew	0	2.70	NS
	Backward spiral horns	9.09	13.51	NS
	Spiral horns facing forward	0	24.32	NS
	Stumps	45.45	0	S (*P <* 0.001)

*S* Significant, *NS* Not significant

The sexual dimorphism was observed for the horn presence and the chamfer profile (Table 3). Indeed, all males were horned against only 6.75% of horned females among which 45.45% were in the form of stumps.

### Molecular genetic diversity

The number of alleles (*Na*), the allelic richness (*AR*), the expected (*He*) and observed (*Ho*) heterozygosities per locus and per DS subpopulation (region) are presented in Table 4. The 12 microsatellite loci used were polymorphic and a total of 89 alleles were detected. The allelic diversity was characterized by the number of alleles ranging from 3 (MAF214) to 10 (MAF10), with an average of 7.42 ± 2.19. The allelic richness estimated using rarefaction method ranged from 2.57 (SRCRSP1) to 4.49 (ILSTS5), with an average of 3.59 ± 0.67. Subpopulations from Bafatá and Gabú had higher genetic diversity with *He* values of 0.716 ± 0.089 and 0.697 ± 0.094, respectively compared to those from Oio (0.655 ± 0.143) and Cacheu (0.651 ± 0.157) regions. Cacheu Djallonké subpopulation presented the smallest diversity index. In Bafatá, Gabú and Cacheu regions, the average observed heterozygosities were lower than the expected heterozygosities under Hardy-Weinberg Equilibrium (HWE).

**Table 4:** Number of alleles (*Na*), allelic richness (*AR*), expected (*He*) and observed (*Ho*) heterozygosities per loci in the four subpopulations of Djallonké sheep

Loci		Bafatá	Cacheu	Gabú	Oio	All populations
**ILSTS5**	***Na***	6	5	8	6	8
	***AR***	4.18	4.15	4.85	4.16	4.49
	***He***	0.794	0.804	0.845	0.788	
	***Ho***	0.692	0.714	0.895	0.938	
**OarCB226**	***Na***	7	5	6	6	8
	***AR***	3.23	3.52	3.39	3.24	3.37
	***He***	0.591	0.706	0.643	0.598	
	***Ho***	0.591	0.800	0.556	0.625	
**OarFCB193**	***Na***	7	5	7	6	9
	***AR***	4.11	3.49	4.05	4.22	4.03
	***He***	0.774	0.701	0.760	0.791	
	***Ho***	0.500	0.800	0.800	0.652	
**OarFCB304**	***Na***	7	5	6	6	9
	***AR***	4.04	3.43	3.97	2.46	3.68
	***He***	0.780	0.664	0.777	0.369	
	***Ho***	0.654	0.333	0.704	0.333	
**ILSTS11**	***Na***	3	3	4	5	6
	***AR***	2.40	2.69	2.83	2.76	2.76
	***He***	0.574	0.549	0.627	0.590	
	***Ho***	0.200	0.533	0.316	0.458	
**MCM140**	***Na***	7	6	7	6	9
	***AR***	4.49	4.25	4.40	3.80	4.29
	***He***	0.817	0.802	0.809	0.715	
	***Ho***	0.842	0.933	0.815	0.708	
**OarJMP58**	***Na***	4	4	3	5	8
	***AR***	4.00	2.70	3.00	3.20	3.49
	***He***	0.750	0.487	0.733	0.693	
	***Ho***	0.750	0.467	0.400	0.783	
**SRCRSP1**	***Na***	4	3	3	3	4
	***AR***	2.76	2.09	2.62	2.63	2.57
	***He***	0.535	0.301	0.553	0.531	
	***Ho***	0.300	0.200	0.630	0.583	
**MAF214**	***Na***	3	3	3	3	3
	***AR***	2.82	2.65	2.79	2.52	2.70
	***He***	0.634	0.545	0.614	0.479	
	***Ho***	0.500	0.667	0.482	0.609	
**MAF65**	***Na***	5	4	4	4	6
	***AR***	3.27	3.26	3.16	3.26	3.25
	***He***	0.690	0.692	0.698	0.699	
	***Ho***	0.808	0.667	0.667	0.652	
**MAF70**	***Na***	8	7	10	8	10
	***AR***	3.51	4.98	4.25	4.43	4.35
	***He***	0.696	0.860	0.750	0.788	
	***Ho***	0.560	0.800	0.769	0.652	
**OarCP34**	***Na***	7	4	5	7	9
	***AR***	4.01	3.41	4.04	4.49	4.10
	***He***	0.736	0.699	0.783	0.824	
	***Ho***	0.600	0.733	0.692	0.913	
**Mean ± SD**	***Na***	5.67 ± 1.78	4.50 ± 1.24	5.50 ± 2.24	5.42 ± 1.51	7.42 ± 2.19
**Mean ± SD**	***AR***	3.57 ± 0.67	3.39 ± 0.80	3.61 ± 0.73	3.43 ± 0.76	3.59 ± 0.67
**Mean ± SD**	***He***	0.697 ± 0.094	0.651 ± 0.157	0.716 ± 0.089	0.655 ± 0.143	0.680 ± 0.032
**Mean ± SD**	***Ho***	0.583 ± 0.192	0.637 ± 0.215	0.644 ± 0.176	0.659 ± 0.169	0.631 ± 0.033

*SD* Standard Deviation

Table 4 Number of alleles (*Na*), allelic richness (*AR*), expected (*He*) and observed (*Ho*) heterozygosities per loci in the four subpopulations of Djallonké sheep.

The effective Ae, the Polymorphic Information Content (PIC) and the F-Statistics (*F*_*IT*_*, F*_*ST*_*, F*_*IS*_) according to Weir and Cockerham (1984) for all the microsatellite markers analyzed over the four DS subpopulations are presented in Table 5. The effective Ae varied from 2 (SRCRSP1) to 5.24 (ILSTS5) with an average of 3.52 ± 1.04. SRCRSP1 locus was the lowest informative with a PIC of 0.45 while ILSTS5 locus presented the highest value of PIC (0.78) and the average value was 0.65 ± 0.11.

**Table 5:** Effective number of alleles (Ae), Polymorphism Information Content (PIC) and the F-Statistics (*F*_*IT*_*, F*_*ST*_*, F*_*IS*_) according to Weir and Cockerham (1984) for 12 microsatellite markers analyzed in four Djallonké sheep subpopulations

Loci	Ae	PIC	***F***_***IT***_	***F***_***ST***_	***F***_***IS***_	_***ST***_
ILSTS5	5.24	0.7823	−0.006	0.009	−0.015	0.031
OarCB226	2.66	0.5888	0.005	−0.000	0.005	0.019
OarFCB193	4.11	0.72	0.086	−0.001	0.087*	0.021
OarFCB304	3.33	0.6622	0.262	0.091	0.188*	0.085
ILSTS11	2.44	0.5142	0.335	0.003	0.333**	0.036
MCM140	4.69	0.758	−0.022	0.016	−0.038	0.029
OarJMP58	3.46	0.6581	0.165	0.169	−0.005	0.152
SRCRSP1	2.00	0.4491	0.075	0.006	0.070	0.028
MAF214	2.34	0.5098	0.047	0.003	0.045	0.020
MAF65	3.22	0.6301	−0.015	− 0.002	− 0.012	0.015
MAF70	4.38	0.7441	0.122	0.020	0.104*	0.036
OarCP34	4.37	0.7384	0.062	0.015	0.048	0.031
**Means ± SD**	**3.52 ± 1.04**	**0.65 ± 0.11**	**0.090 ± 0.031**	**0.029 ± 0.016**	**0.063 ± 0.029**	**0.043**

*SD* Standard Deviation

The mean values of *F*_*IT*_*, F*_*ST*_*, F*_*IS*_ were 0.09, 0.029 and 0.063, respectively. Values of *G*_*ST*_ ranged from 0.015 for MAF65 to 0.152 for OarJMP58, with a mean of 0.043 showing that the gene variation among subpopulations is still low. The *F*_*ST*_ value (0.029) showed that most of the total genetic variation corresponds to differences among individuals within subpopulation (97.10%) and only 2.90% result from differences among subpopulations.

The overall estimate of *F*_*IS*_ was 0.063 ± 0.029. The subpopulation-wise *F*_*IS*_ estimates were significantly (*P <* 0.01) greater than zero in Bafatá and Gabú subpopulations, suggesting a deviation from HWE (Table 6). The exact tests also showed a significant deviation from HWE for some markers in the different subpopulations.

**Table 6:** *F*_*IS*_ values in the four Djallonké Sheep subpopulations

Loci	Bafatá	Cacheu	Gabú	Oio
ILSTS5	0.133	0.116	−0.061	− 0.197
OarCB226	0.000	−0.139	0.138	−0.047
OarFCB193	0.362*	−0.147	−0.054	0.179**
OarFCB304	0.164	0.507*	0.096	0.098
ILSTS11	0.664*	0.030	0.503**	0.226
MCM140	−0.032	−0.170	−0.007	0.009
OarJMP58	0.000	0.044	0.484	−0.133
SRCRSP1	0.445**	0.344	−0.142	−0.101
MAF214	0.215*	−0.233	0.219	−0.278
MAF65	−0.174	0.038	0.046	0.068
MAF70	0.199	0.072	−0.027	0.176*
OarCP34	0.187	−0.051	0.118	−0.111
**All loci**	0.169***	**0.022**	0.107**	**−0.006**

*P < 0.05, ** P < 0.01, ***P < 0.001

The overall differentiation level of the subpopulations was very low (*F*_*ST*_ = 0.029 ± 0.016). Among the four subpopulations, the lowest genetic distance was observed between Bafatá and Gabú subpopulations (0.0406) and the highest between Bafatá and Cacheu subpopulations (0.1412). The genetic distances and the genetic identity according to Nei (1978) are summarized in Table 7.

**Table 7:** Genetic distance (below the diagonal) and genetic identity (above the diagonal) according to Nei (1978)

	Bafatá	Cacheu	Gabú	Oio
**Bafatá**	–	0.8683	0.9603	0.9107
**Cacheu**	0.1412	–	0.9097	0.8940
**Gabú**	0.0406	0.0946	–	0.9440
**Oio**	0.0936	0.1121	0.0576	–

**Fig. 3:**
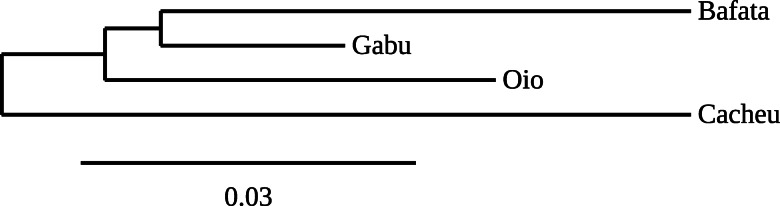
Unrooted neighbor-joining tree depicting the relationship of four subpopulations of Djallonké Sheep of Guinea-Bissau using Nei’s (1978) genetic distances

From the unrooted neighbor-joining tree constructed using the genetic distances (Fig. 3), the subpopulation from Cacheu region relatively differed from the three other subpopulations.

### Genetic structure of subpopulations by factorial correspondence analysis

**Fig. 4:**
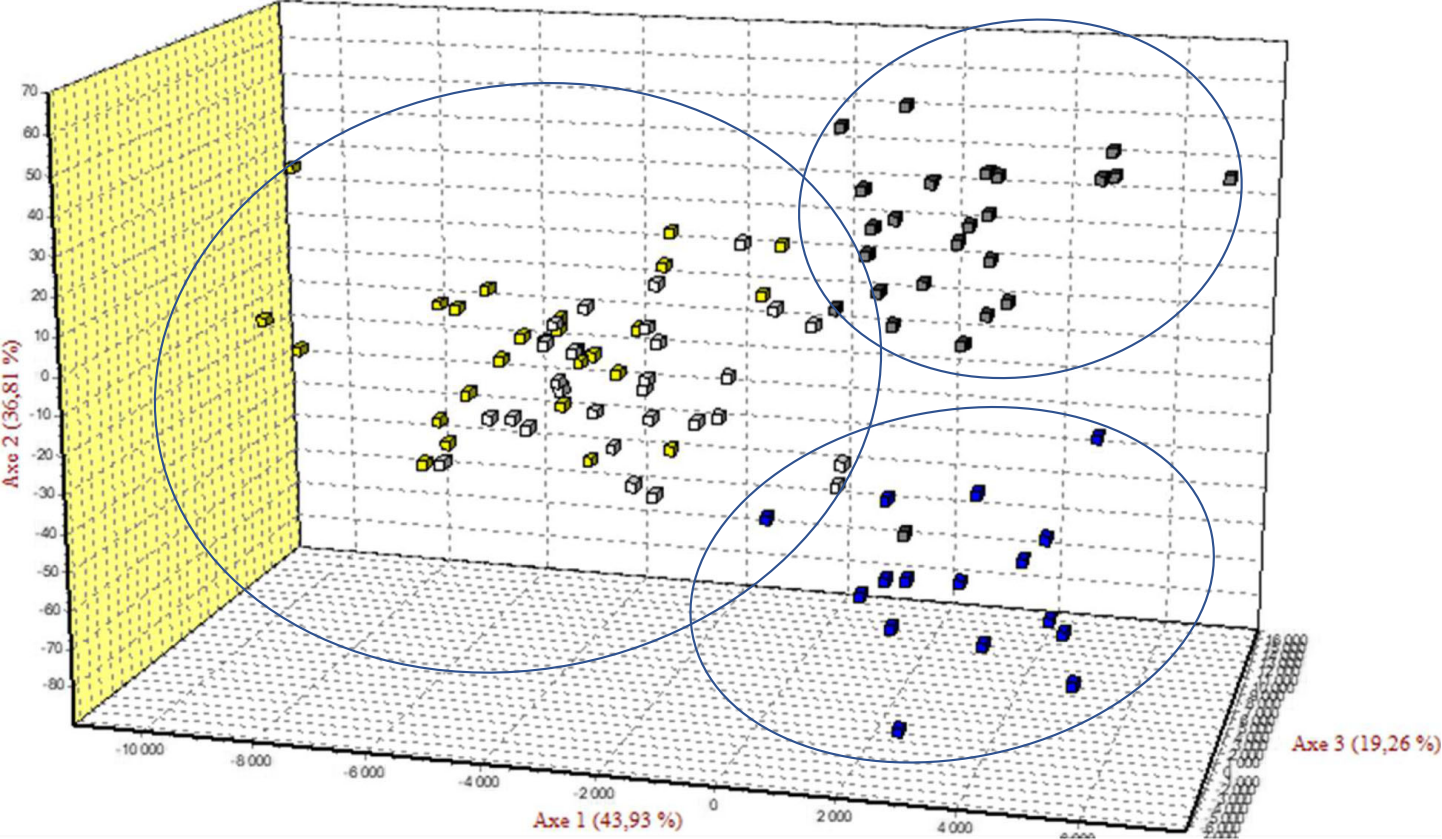
Factorial correspondence analysis. Yellow: Bafatá; Blue: Cacheu; White: Gabu; Grey: Oio. Axis 1 isolated Gabú – Bafatá and Oio from Cacheu while Axis 2 delimited Oio and Cacheu

The factorial correspondence analysis (Fig. 4) clustered the studied population in three groups: group 1 with Bafatá and Gabú subpopulations, group 2 with predominantly Oio subpopulation and group 3 with the Cacheu subpopulation. Although the *F*_*ST*_-pairwise values were very low, the FCA allowed to represent the different subpopulations. The factorial axis 1 (43.93%) separates Bafatá and Gabú subpopulations from those of Oio and Cacheu while the factorial axis 2 (36.81%) isolated Oio subpopulation from Cacheu subpopulation.

**Fig. 5:**
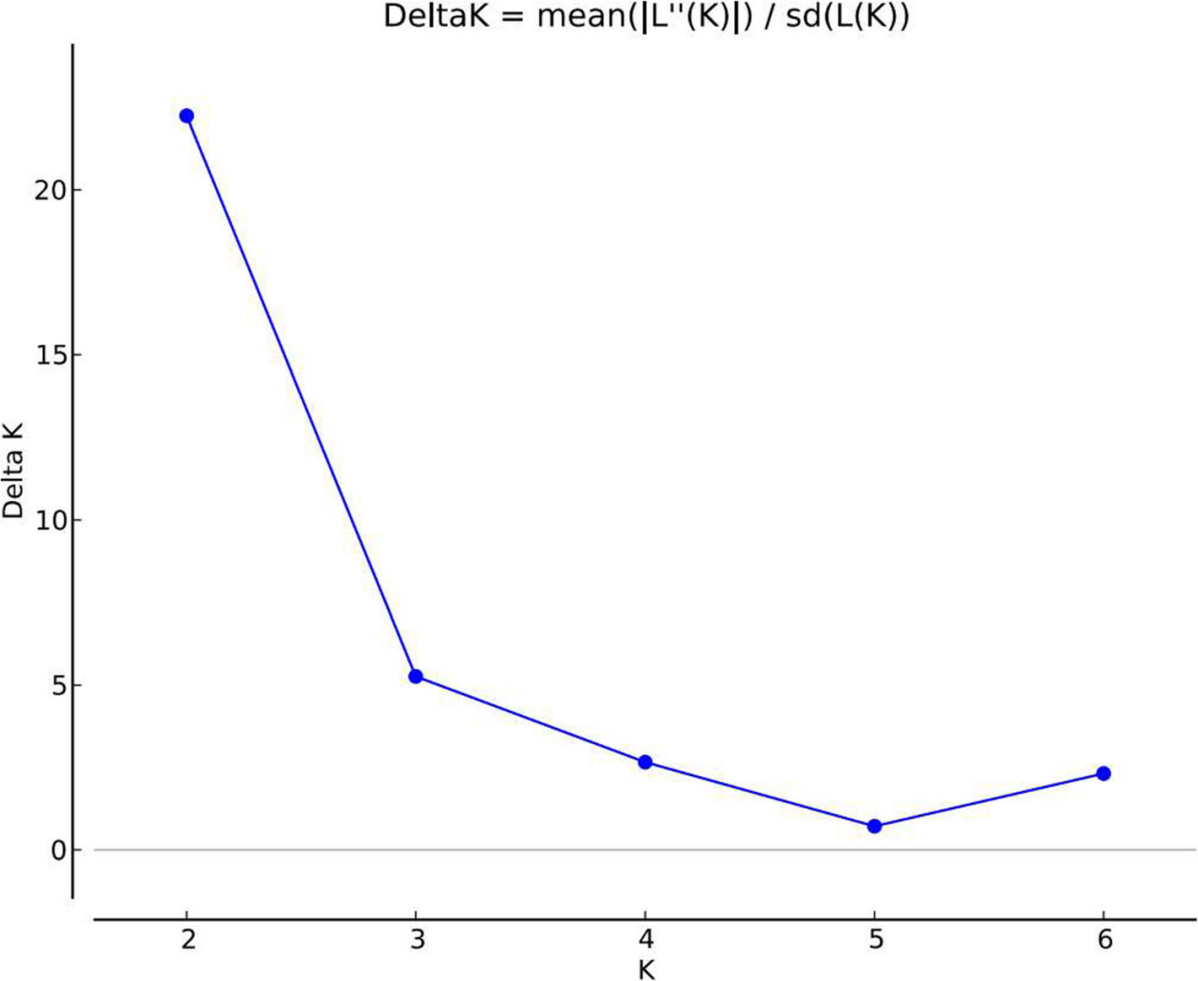
Plots for detecting the number of K groups that best fit the data (Assumption: No Admixture Model and Independent Alleles frequencies)

Using Bayesian approach implemented in Structure Software and Evanno method [9], the number of K groups that best fit the data is detected between 2 and 3 (Fig. 5).

**Fig. 6:**
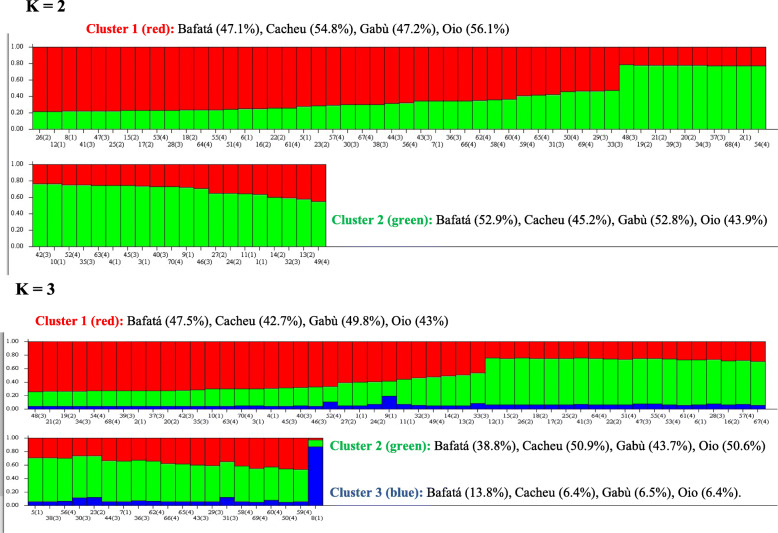
Population structure assessed by Structure software. Each individual is represented by a vertical bar, often partitioned into colored segments with the length of each segment representing the proportion of the individual’s genome from K = 2 to 3 ancestral populations (Animals for which more than 2 loci were not amplified were removed from this analysis)

Assuming K = 2, Cacheu and Oio clustered in the group 1 with 54.8 and 56.1% respectively while Bafatá and Gabù clustered in group 2 with 52.9 and 52.8% respectively. At K = 3, Bafatá and Gabù subpopulations with 47.5 and 49.8% respectively remained in the cluster 1, Cacheu (50.9%) and Oio (50.6%) in the Cluster 2 and the four subpopulations were in the cluster 3 with 13.8% for Bafatá, 6.4% for Cacheu, 6.5% for Gabù and 6.4% for Oio (Fig. 6).

## Discussion

### Morphological diversity

#### Quantitative characters

DS in Guinea-Bissau can be classified into three “genetic types” associated to three the “large animals” in the Bafatá region, “intermediate traits” for sheep in the Gabù and Oio regions and “small animals” in the Cacheu region. Indeed, the average values of the quantitative characters (CG, CD, HW, BL, EL and TL) of the Bafatá DS subpopulation were significantly higher than those obtained in the Gabú, Oio and Cacheu regions. This gradient in the size of the morphological traits could be explained by the differences in the agro-ecological conditions, the farming practices and genetic background. In fact, the agro-ecological area of the North-East, which includes the Bafatá, Gabú and Oio regions, is characterized by savannah trees and clear forests, which offer rich natural pastures to pastoralists who are Fulani and Mandingos. Moreover, the livestock is dominated by ruminant species. Contrariwise, in the North-West agro-ecological zone including the Cacheu region, ruminant species (sheep, goat and cattle) are mainly raised for ritual ceremonies by breeders who are rather animistic [10]. In addition, this zone is covered with wooded savannahs and dense forests hardly accessible by animals, hence the predominance of the sedentary system in the Cacheu region. At the cultural level, Bafatá region is mainly populated by Fula-speaking people, practicing the Muslim religion and traditionally attached to animal husbandry compared to the other regions (Cacheu and Oio) where the populations are strongly Christianized and more attached to pig farming. The Bafatá region is also a large area of ruminant species concentration during the transhumance period and hosts the most important livestock market in the country. This region generally receives animals from Gabú and both Gabú and Bafatá regions have more than 70% of the country’s ruminant livestock [4]. During the dry season (November to May), ruminants from the Gabú region migrate to the Bafatá and Oio regions [11].

Sheep from the Cacheu region had the smallest size in the study area. In fact, Cacheu is one of the regions of the North-West agro-ecological zone with high humidity favorable to parasitism and vectors of pathogens such as tsetse flies which transmit the trypanosomes causing African animal trypanosomosis.

DS subpopulations of the Gabú and Oio regions were highly heterogeneous with an “intermediate genetic type”, probably due to the introduction of improving rams in these regions in the past [12]. This heterogeneity is observed not only between regions but also within region (Fig. 1). The effect of the agro-ecological zone on the morphological types of ruminants, especially sheep, has been previously reported in Côte d’Ivoire in DS [13], in Senegal with Peul-peul (Fulani) sheep [14] and in Togo in Vogan Sheep and DS [15]. A recent morphobiometric characterization of DS in the sudano-guinean zone of Cameroon revealed three genetic types [16] as observed in the present study in Guinea-Bissau. In Burkina Faso, Traoré et al. [17] described a sheep population named “Mossi sheep” which is a savannah DS found in an agro-ecological zone between the sudano-sahelian zone and the sudano-guinean zone with an “intermediate type” between DS and sahelian sheep.

The average values of HW obtained (55.67 ± 4.16 cm for the Bafatá region, 54.67 ± 3.44 cm for the Oio region, 53.44 ± 2.85 cm for the Cacheu region and 53.23 ± 3.77 cm for Gabú region) are closed to those reported by Dayo et al. [15] in DS in Togo (HW = 54.63 ± 8.23 cm; BL = 58.47 ± 6.30 cm and CG = 74.72 ± 8.28 cm) and Sangaré [18] in DS in West Africa and Gueye [19] in Senegal. Similar results have also been reported in other populations of DS in Ghana (HW = 57.06 ± 0.28 cm; BL = 54.87 ± 0.35 cm and CG = 69.19 ± 0.41 cm) by Birteeb et al. [20] and Asamoah-Boaheng and Sam [21] and in Côte d’Ivoire (HW = 59.60 ± 5.40 cm; BL = 57.80 ± 5.40 cm and CG = 70.80 ± 6.50 cm) by N’Goran et al. [13]. However, the values of the present study were higher than those previously reported by Hadzi [22] in DS in Togo and in Guinea-Bissau [8]. These results could be explained by the differences of climatic conditions of the agro-ecological zones in which these studied populations are bred, the study periods of the year (season effect), the farming systems or the genetic variability that could be observed between DS populations across the countries. It has been reported the existence of two sub-categories of DS [23, 24] and DS of savannah are larger than those of forest zones [25], demonstrating once more the effect of the agro-ecological zone on the morphological type of this sheep breed.

The tail of the DS is thin and relatively long. The average TL (28.06 ± 4.36 cm) is similar to those reported by N’Goran et al. [13] in DS in Côte d’Ivoire (24.70 ± 3.40 cm) and in Togo (27.47 ± 8.05 cm) [15]. This TL is longer than those reported in the DS (West African Dwarf) by Gbangboche et al. [25] in Benin (17 cm), in Nigeria (19.42 ± 0.63 cm) [26] but shorter than those of the Sahelian sheep (48.20 ± 5.37 cm) and Vogan sheep from Togo (45.24 ± 6.23 cm) [15].

Concerning the ear length, the value obtained (9.85 ± 1.12 cm) is similar to value reported by Gbangboche et al. [25] in West-Africa, who found that DS has small ears, about 10 cm. However, the value in the present study is lower than those reported in DS in West Africa:13.03 ± 0.39 cm in Nigeria [26], 11.61 ± 2.61 cm in Togo [15]) and in the Peul-peul (Fulani) sheep (13.30 ± 1.20 cm) in Senegal [14]; and significantly shorter than those recorded in Vogan sheep (18.45 ± 2.08 cm) and Sahelian sheep (21.63 ± 2.48 cm) [15]. No sexual dimorphism was observed for this trait contrary to Gueye [19] who showed that male sheep and goats had slightly longer ears than females in Senegal.

#### Qualitative characters

The coat color pattern in DS in Guinea-Bissau is dominated by the uniform white pattern and the spotted white and brown / fawn pattern in all regions. In the Bafatá region, the frequency of the spotted pattern is higher than in the other three regions. Indeed, for the Muslim populations in Bafatá and Gabú regions, the rams are preferentially slaughtered while the uniform white or spotted ewes are kept for the reproduction in order to have the offspring with white coat color. This explained the presence of only few rams in most of herds. The higher proportion of animals with uniform white color pattern could also be due to a strong selection of animals expressing the white coat color to meet the livestock market demands (higher price than other coat colors) and the cultural preference in the country (religious sacrifices or gifts during baptism celebrations and the “Eid El-Kebir” (Tabaski) celebration or for the dowry). The preferences for the coat color of animals differ from one society to another. For example, in southern Ethiopia, red coat color for ewes is the most suitable for market demands [27]. In Côte d’Ivoire, the DS had at 55.00% patchy white-black coat color compared to 24.00% uniform white coat [13], and only 5.88% of the DS were white in southern Togo [15]. This diversity for coat color in DS in West Africa is linked to the choices made by the societies in which these animals are raised. In Ferlo zone in Senegal, the dominant coat color of the Peul-peul sheep has evolved from patchy (white-black or white-red/fawn) [19] to spotted of white and black / red/fawn [28].

The ears of DS in Guinea-Bissau are mostly erected horizontally and only 2.67% of animals in the Bafatá region had slightly drooping ears. These results agree with those of Dayo et al. [15] in DS from Togo (86.27%) and N’Goran et al. [13] in Côte d’Ivoire (87.00%). Drooping ears in DS are considered to be the result of Sahelian sheep genes introgression [13, 15]. Thus, the presence of animals with slightly drooping ears in the Bafatá region (2.67%) could be explained by crosses occurred with Sahelian sheep from neighboring countries, especially from Senegal.

Sexual dimorphism has been observed for the presence of horns with only 6.75% females horned in our study. This proportion is higher than the 2.30% often reported for ewes wearing horns (most are stumps); but lower than the 14.60% of Mossi ewes carrying horns in Burkina Faso [17]. The horns are developed for rams and absent or in stumps in ewes. In the current study, the most of horned ewes were from the regions of Bafatá and Oio where small ruminants and cattle move during the transhumance in the dry season [11]. Horned ewes are thought to have come from crossing with transhumant animals. It is important to highlight that in half of these ewes, the horns are in stumps.

The horn shapes were significantly different according to the zone: horns laterally straight were the most observed in Bafatá, Gabú and Oio regions while spiral horns facing forward predominated in the forest and humid Cacheu region similarly to the one reported by Dayo et al. [15] in the south of Togo.

### Molecular genetic diversity

The current study provides the first information on molecular genetic characterization of DS in Guinea-Bissau and is complementary to the morphological characterization of this breed. This study presents a comprehensive genetic analysis of DS, the assumed only sheep breed of Guinea-Bissau, from four administrative regions covering two agro-ecological zones. The genetic diversity of subpopulations was influenced by the socio-cultural practices and agro-ecological zones. Similar observations were reported by prior studies in West African DS [29]. Indeed, these authors had reported that Malian, Gambian and eastern Guinean DS populations had higher genetic diversity than those from Senegal and southern and western Guinean using expected heterozygosity (*He*) and the mean number of alleles (*Na*). Based on the *He,* Cacheu and Oio DS subpopulations would be closer to Senegalese, Gambian southern and western Guinean populations while Bafatá and Gabú DS presented similar expected heterozygosities to Malian and eastern Guinean DS. The *Na* in the current study (7.42 ± 2.19) was similar to those obtained by Wafula et al. [29] in Guinean and Malian DS and Agaviezor et al. [26] in West African Dwarf sheep in Nigeria. However, the allelic richness (adjusted mean number of alleles) values were lower than those reported by Wafula et al. [29] and Agaviezor et al. [26] and probably due to the small sample size used for genotyping in our study.

### Genetic structure of the population

Using different population differentiation parameters (*F*_*ST*_*, G*_*ST*_, genetic distance, genetic identity) and representation (NJ Tree and FCA), our results showed that the population differentiation over the 4 subpopulations is very low since the multi-locus *F*_*ST*_ and *G*_*ST*_ values indicated that only 2.9 and 4.3% respectively of the total genetic variation were due to the subpopulation differences. The remaining 97.1 for *F*_*ST*_ and 95.7 for *G*_*ST*_ corresponded to differences between individuals within the subpopulations. These values were lower than those (8.8% for *F*_*ST*_ and 12% for *G*_*ST*_) reported by Agaviezor et al. [26] in four sheep populations in Nigeria (Udah, Balami, Yankasa and West African Dwarf sheep also known as DS). Even though the genetic differentiation observed between the four DS subpopulations in Guinea-Bissau was low, the current study pointed that the subpopulation from Cacheu region slightly differs from those in Gabú, Bafatá et Oio regions. Indeed, these three subpopulations are genetically close even though they come from geographically different locations. This similarity is shown by: *i)* the high genetic identity (from 0.9603 to 0.9017) of the three subpopulations while this value decreased to 0.8683 between Bafatá and Cacheu subpopulations*, ii)* the low genetic distances between the three subpopulations. The closest Nei’s [30] unbiased measures between Bafatá and Gabú, and the farthest between Bafatá and Cacheu may be due not only to their geographical locations but also to the breeding systems, the presence of the livestock market in Bafatá and the cultural behavior of the breeders in the different regions. Ira et al. [5] reported that Bafatá, Gabú and Oio regions had 95.88% of the sheep population of Guinea-Bissau and breeders practice transhumance breeding system, mixing cattle and sheep while in Cacheu region the breeding system is rather sedentary in association with agriculture (production of mangrove rice, sorghum, millet, beans, peanuts and cashew). The Bayesian approach implemented in STRUCTURE program detected the number of K that best fit the data between 2 and 3, suggesting an introgression of the Djallonké sheep of Guinea Bissau by an exotic sheep or the existence of “ecotypes”. The two subpopulations from the eastern regions (Gabù and Bafatà) were separated from the western subpopulations (Cacheu and Oio) at K = 2. The heterogeneity of the DS in Guinea-Bissau is shown with K = 3. The molecular study on DS population of Guinea-Bissau confirmed the results obtained from phenotypic study.

Further investigations extended to other regions of Guinea-Bissau and other sheep breeds are required to determine the origin of the admixture and the existence of ecotypes of Djallonké sheep in this country.

## Conclusions

In this primary phenotypic characterization of the DS in Guinea-Bissau, three genetic types of animals were distinguished, namely the largest animals in Bafatá, the smallest animals in Cacheu and the type with intermediate traits of animals in Gabú and Ohio. The values of the quantitative characters of the sheep of the region of Bafatá were significantly higher than those of the other regions. The molecular study confirmed the existence of three genetic groups in the DS population in Guinea-Bissau that could be related more to breeding system than a genetic differentiation which was very low. The current study provides sufficient data that could be used to develop strategies for the sustainable and efficient management of animal genetic resources in general and specifically of sheep genetic resources in Guinea-Bissau. To complete the morphological and molecular characterization reported in the current study, it would be necessary to collect and analyze the demographic parameters and the zootechnical data of the DS population in Guinea-Bissau.

## Methods

### Study area and population

The study was conducted in four administrative regions which are the largest agro-pastoral areas in Guinea-Bissau: Bafatá, Gabú, Cacheu and Oio. These four regions cover two agro-ecological zones [31]:

- the North-East area comprising the regions of Gabú, Bafatá and Oio: characterized by a Sudanese climate with two distinct seasons: a dry season between November and May, and a rainy season from June to October. The annual rainfall ranges from 1200 to 1500 mm over an average of 107 days. The rate of evapotranspiration is 2507 mm and the annual average temperature is 27.4 °C. Most of the soil is tropical iron and iron. However, hydromorphic soils derived from marine alluvium are found in the shallows, basins of rivers and rivers. The vegetation consists of wooded savannahs and clear forests dotted with grasses that provide excellent natural grazing for animals. Livestock is dominated by ruminants and associated with the cultivation of maize, plains rice, sorghum, millet, cotton, groundnuts and cashew nuts. DS is the only sheep breed used in these regions. Animal breeding is practiced by ethnic Peulh populations and Mandingoes with Muslim religious dominance (5);

- the North-West area comprising the regions of Cacheu, Bissau and Biombo: moderately wet and warm Guinean maritime climate with 1500 1877 mm of average rainfall over 112 days. The average annual temperature is 26.6 °C and the evapotranspiration is 137 mm [31]. This area offers good opportunities for diversified agricultural production. The soils are sandy-clay and hydromorphic. The vegetation is made up of wooded savannahs and dense forests. Livestock is dominated by pigs and poultry. The reduced size herds of ruminants are also met. Ruminants and poultry are much more used for traditional rituals than for sale at the market. This system is practiced by animist populations such as Pepels, Balantes, Manjaques, Diolas, Mancanhes and Bijagós. The husbandry is associated with the cultivation of low-lying rice, sorghum, millet, groundnuts, sweet potatoes, cassava and cashew nuts.

Animals belonging to Djallonké Sheep breed, both adult males and females were included in the study. Data collection was carried out between April and October 2017.

Morphobiometric data (qualitative and quantitative traits) were collected through single visits (primary characterization) in the different herds. A total of 200 animals were chosen in the four administrative regions: 75 animals in the Bafatá region, 25 in the Cacheu region, 60 in the Gabú region and 40 in the Oio region. The herds were chosen after sensitization of the breeders and their agreement. In each herd, the least related adult animals were chosen. Locations of the animal sampling have been included in Supplementary Fig. S1.

### Description of animal morphological characters and body measurements

Body measurements (quantitative variables) concerned: (i) the Height at the Withers (HW), the Chest Depth (CD) and the Body Length (BL) using a sliding ruler; (ii) the Chest Girth (CG), Ear Length (EL), the Horn Length (HL), the Interval Length between the roots of the two Horns in males (ILH) measured between the roots of the two horns and the Tail Length (TL) were determined using a measurement tape. Body parameters measurements were taken early in the morning to avoid changing the animal’s conformation after consuming water and food.

The morphological characteristics (qualitative variables) related to the sex of the animal (male / female), the type of melanin (eumelanin, phaeomelanin, absence of pigment), the coat color pattern, the coat color, the ear orientation, the facial (chamfer) profile, the presence or absence of horns and the shape of the horns were described using visual criteria by simple observation of the interviewers following the elements of the guidelines developed for the study using the guidelines of the Food and Agriculture Organization for the phenotypic characterization of Animal Genetic Resources [7].

### DNA extraction, polymerase chain reaction and fragment analysis

Blood samples were collected on 92 unrelated animals: 26 animals in the Bafatá region, 15 in the Cacheu region, 27 in the Gabú region and 24 in the Oio region. Farmers were interviewed in detail to ensure unrelatedness among the sampled individuals. About 5 ml of whole blood samples were collected after jugular venipuncture in EDTA coated vacutainer tubes. Genomic DNA was extracted using Commercial PROMEGA Wizard purification kit. A total of 12 microsatellite markers chosen among those recommended by the FAO-ISAG consortium [32] were used to genotype all the individuals (Table 8). The forward primer for each locus was labelled with one of the four fluorescent dyes FAM, VIC, NED and PET (Applied Biosystems, USA). Multiplexed polymerase chain reaction was performed with a total reaction volume of 12 μl containing 5 μl of mix primers of multiplex, 5 μl of mix of other reagents (Buffer, MgCl_2_, Taq polymerase) and 2 μl of DNA. The following thermal conditions, 94 °C for 15 min, followed by 40 cycles of 94 °C for 30 s, specific annealing temperature (58 °C and 60 °C according to the multiplex) for 1 min 45 s and 72 °C for 1 min 30 s and a final extension at 72 °C for 15 min was used for sample amplification by PCR. The amplified PCR products containing different dyes were then electrophoresed in four multiplexes (Table 8) in an automated DNA sequencer along with LIZ600 (Applied Biosystems, USA) as an internal lane control. The allele size data for each sample was generated using GENEMAPPER software version 5.

**Table 8:** Characteristics of the sheep microsatellite markers

Microsatellite	Primers	Sequences of primers	Nucleotide pattern	Number of chromosome	Hybridization temperature (°C)	Multiplex	Theoretical size
OarJMP58	Forward	GAAGTCATTGAGGGGTCGCTAACC	Di	OAR 26	58	1	145–169
	Reverse	CTTCATGTTCACAGGACTTTCTCTG					
MAF214	Forward	GGGTGATCTTAGGGAGGTTTTTGGAGG	Di	OAR 16	58	2	174–282
	Reverse	AATGCAGGAGATCTGAGGCAGGGACG					
ILSTS5	Forward	GGAAGCAATGAAATCTATAGCC	Di	OAR 7	55	3	174–218
	Reverse	TGTTCTGTGAGTTTGTAAGC					
MAF65	Forward	AAAGGCCAGAGTATGCAATTAGGAG	Di	OAR15	60	2	123–127
	Reverse	CCACTCCTCCTGAGAATATAACATG					
OarFCB193	Forward	TTCATCTCAGACTGGGATTCAGAAAGGC	Di	OAR 11	54	3	174–218
	Reverse	GCTTGGAAATAACCCTCCTGCATCCC					
OarFCB304	Forward	CCCTAGGAGCTTTCAATAAAGAATCGG	Di	OAR 19	56	3	150–188
	Reverse	CGCTGCTGTCAACTGGGTCAGGG					
ILSTS11	Forward	GCTTGCTACATGGAAAGTGC	Di	OAR 9	55	1	256–294
	Reverse	CTAAAATGCAGAGCCCTACC					
MCM140	Forward	GTTCGTACTTCTGGGTACTGGTCTC	Di	OAR 6	60	1	167–193
	Reverse	GTCCATGGATTTGCAGAGTCAG					
SRCRSP1	Forward	TGCAAGAAGTTTTTCCAGAGC	Di	OAR 13	54	1	116–148
	Reverse	ACCCTGGTTTCACAAAAGG					
OarCP34	Forward	GCTGAACAATGTGATATGTTCAGG	Di	OAR 3	50	4	112–130
	Reverse	GGGACAATACTGTCTTAGATGCTGC					
OarCB226	Forward	CTATATGTTGCCTTTCCCTTCCTGC	Di	OAR 2	60	3	119–153
	Reverse	GTGAGTCCCATAGAGCATAAGCTC					
MAF70	Forward	CACGGAGTCACAAAGAGTCAGACC	Di	OAR 4	60	4	124–166
	Reverse	GCAGGACTCTACGGGGCCTTTGC					

### Data analysis

#### Morphological data analysis

The statistical analysis of the qualitative and quantitative data was done using R 3.5.1 software [33].

For qualitative data, frequencies and proportions were analyzed by region and sex using the Chi-square test.

Means, standard deviations and extreme values (minimum, maximum) were computed for all studied traits. For the quantitative variables following the normal distribution, the comparisons of the means between regions or sexes were computed using parametric tests, in particular the one-way analysis of variance (ANOVA) while for those which did not follow the normal distribution, these means were compared using non-parametric tests (Kruskal-Wallis test, *KW*). Multivariate analysis (principal components analysis, PCA) was used to investigate morphological structure and quantify differences among subpopulations of DS from the four regions using the FactoMiner Package implemented in R software [34].

#### Genotypic data analysis

Allele numbers, allelic richness, the unbiased estimator of Wright’s inbreeding coefficient *F*_*IS*_, *F*_*IT*_, *F*_*ST*_ calculated according to Weir and Cockerham [35] for each locus were determined using FSTAT software version 2.9.4 [36]. The rarefaction approach for the allelic richness estimation uses the frequency distribution of alleles at a locus to estimate the number of alleles that would occur in smaller samples of individuals. It is used to standardize Â to the smallest N in a comparison [37]. Additionally, observed and unbiased expected heterozygosities per locus as well as the factorial correspondence analysis (FCA) were estimated using GENETIX 4.03 (http://www.genetix.univ-montp2.fr). Departures from Hardy–Weinberg equilibrium over all loci were evaluated using Fisher’s method implemented in Genepop v.4.7.2 [38]. The same software was used to perform the score test for Hardy-Weinberg equilibrium [39] per locus using a Markov chain algorithm with 10,000 dememorizations, 200 batches and 5000 iterations per batch. The Hardy-Weinberg equilibrium test measures the difference between the observed numbers of population genotypes and the theoretical genotypic numbers obtained with the Hardy-Weinberg relationship. The effective number of alleles (Ae) and the polymorphic information content (PIC) for each locus were analyzed by using Molkin v. 3.0 software [40]. The genetic identity and genetic distances were calculated using Popgene version 1.31 [41]. The unrooted neighbor-joining tree based on Nei’s (1978) genetic distances was constructed using PHYLIP version 3.698 [42].

To assign individuals to K populations and estimate the posterior distribution of each individual’s admixture coefficient, we used STRUCTURE software 2.3.4 [43] which is a model-based clustering method that utilizes a Monte Carlo Markov Chain. Because genotyping information for the putative parental populations was not available, we hypothesized k parental unknown populations (k varying from 1 to 8 with 10 replicated runs for each K). Analysis was performed with a burn in length of 50,000 followed by 100,000 Markov chain Monte Carlo iterations for each of K using uncorrelated allelic frequencies between the parental populations and an admixture model.

The optimal ‘K’ was identified based on ∆K, the second order rate of change in LnP(D) following the likelihood procedure of Evanno et al. [9] using Structure Harvester (available at http://taylor0.biology.ucla.edu/structureHarvester/). Structure Harvester [44] is a web-based program for collating results generated by the STRUCTURE program to identify the best value of K. The program provides a fast way to assess and visualize likelihood values across multiple values of K and hundreds of iterations for easier detection of the number of genetic groups that best fit the data.

## Supplementary Information

**Additional file 1: Figure S1.** Map (study area) of the origins of Djallonké sheep sub-populations.

## Data Availability

The datasets used and analyzed during the present study are available from the corresponding author on reasonable request. All data used have also been published on. Preview of Morphological and SSR_Genetic data_Djallonke_Sheep - Mendeley Data (https://data.mendeley.com/v1/datasets/p2z3z3sxdg/draft?preview=1). DOI: 10.17632/p2z3z3sxdg.2

## Abbreviations

*Ae*: Effective Allele Number
ANOVA: Analysis of variance
*AR*: Allelic Richness
BL: Body Length
CD: Chest Depth
CG: Chest Girth
DNA: Deoxyribonucleic acid
DS: Djallonké Sheep
EL: Ear Length
FCA: Factorial Correspondence Analysis
*F*_*IS,*_*F*_*IT,*_*F*_*ST*_: F-Statistics indices
*Ho*: Observed heterozygosity
*He*: Expected heterozygosity
HL: Horn Length
HW: Height at the Withers
HWE: Hardy-Weinberg equilibrium
ILH: Interval Length between the roots of the two Horns in males
PCA: Principal components analysis
PIC: Polymorphism Information Content
TL: Tail Length
