# Supplementary figures and images for: Morphological and microsatellite DNA diversity of Djallonké sheep in Guinea-Bissau
Source: BMC Genom Data. 2022 Jan 7;23:3. doi: 10.1186/s12863-021-01009-7 (PMC8740422; doi:10.1186/s12863-021-01009-7)

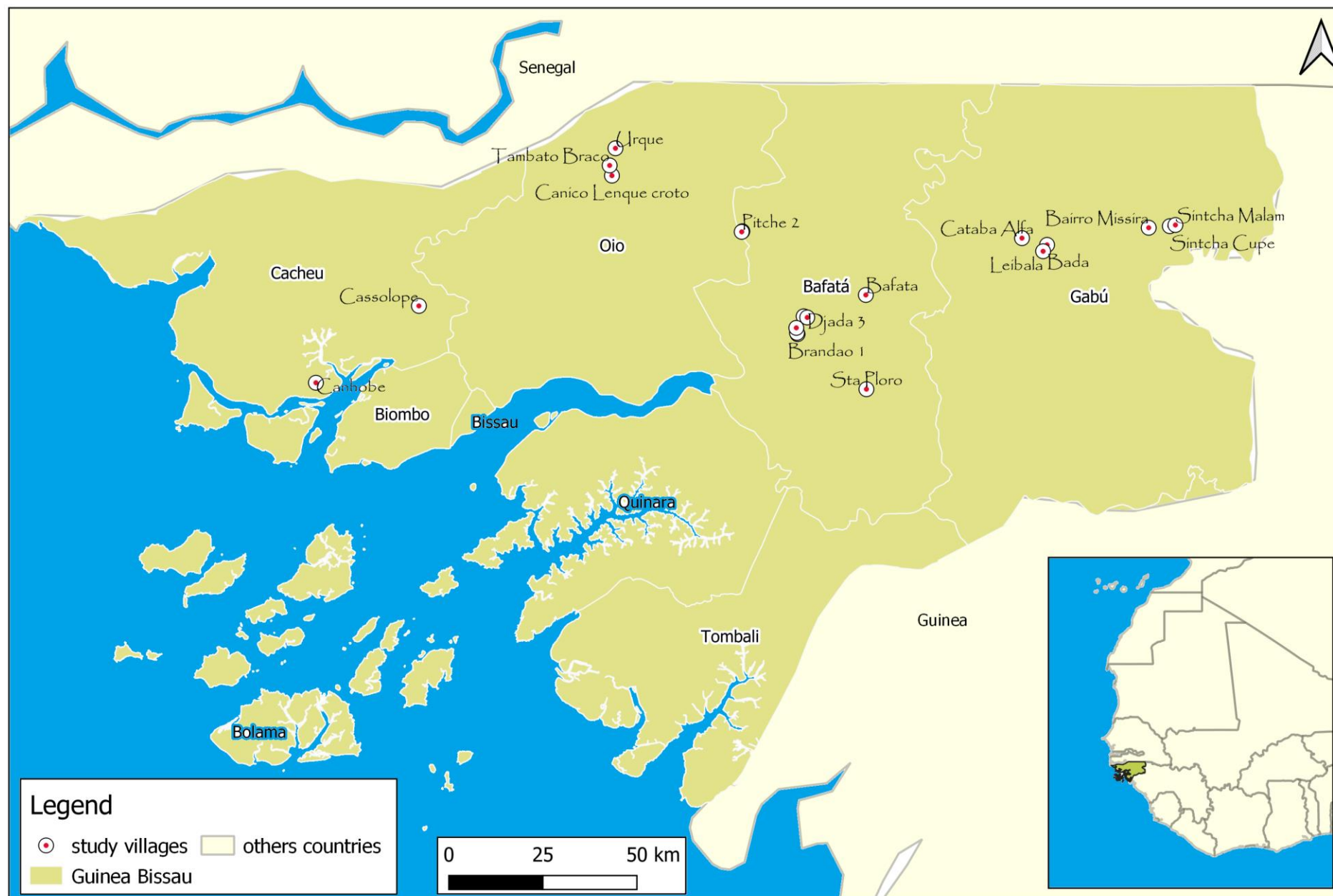

Figure S1 Map of the study area of Djallonké sheep sub-populations

Supplement: Supplementary file 1 — Additional file 1: Figure S1. Map (study area) of the origins of Djallonké sheep sub-populations. [file 12863_2021_1009_MOESM1_ESM.pdf]
